# Hip replacement improves lumbar flexibility and intervertebral disc height — a prospective observational investigation with standing and sitting assessment of patients undergoing total hip arthroplasty

**DOI:** 10.1007/s00264-022-05497-9

**Published:** 2022-07-11

**Authors:** Maximilian Muellner, Zhen Wang, Zhouyang Hu, Sebastian Hardt, Matthias Pumberger, Luis Becker, Henryk Haffer

**Affiliations:** Center for Musculoskeletal Surgery, Charité - Universitätsmedizin Berlin, Corporate Member of Freie Universität Berlin, Humboldt-Universität Zu Berlin, Charitéplatz 1, 10117 Berlin, Germany

**Keywords:** Low back pain, Hip–spine syndrome, Hip arthroplasty, Spinopelvic complex, Spinopelvic function, Lumbar spine

## Abstract

**Purpose:**

The pathogenic mechanism of the hip–spine syndrome is still poorly elucidated. Some studies have reported a reduction in low back pain after total hip arthroplasty (THA). However, the biomechanical mechanisms of THA acting on the lumbar spine are not well understood. The aim of the study is to evaluate the influence of THA on (1) the lumbar lordosis and the lumbar flexibility and (2) the lumbar intervertebral disc height.

**Methods:**

A total of 197 primary THA patients were prospectively enrolled. Pre- and post-operative biplanar stereoradiography was performed in standing and sitting positions. Spinopelvic parameters (lumbar lordosis (*LL*), pelvic tilt, sacral slope, pelvic incidence), sagittal spinal alignment (sagittal vertical axis, *PI*-*LL* mismatch (*PI*-*LL*)) and lumbar disc height index (*DHI*) for each segment (L1/2 to L5/S1) were evaluated. The difference between standing and sitting *LL* (∆*LL* = *LL*_standing_ − *LL*_sitting_) was determined as lumbar flexibility. Osteochondrosis intervertebralis was graded according to Kellgren and Lawrence (0–4), and patients were assigned to subgroups (mild: 0–2; severe: 3–4).

**Results:**

Lumbar flexibility increased significantly after THA (pre: 22.04 ± 12.26°; post: 25.87 ± 12.26°; *p* < 0.001), due to significant alterations in *LL* in standing (pre: 51.3 ± 14.3°; post: 52.4 ± 13.8°; *p* < 0.001) and sitting (pre: 29.4 ± 15.4°; post: 26.7 ± 15.4°; *p* = 0.01). ∆*LL* increased significantly in both subgroups stratified by osteochondrosis (pre/post: Δ*LL*_mild_: 25.4 (± 11.8)/29.4 ± 12.0°; *p* < 0.001; Δ*LL*_severe_: 17.5 (± 11.4)/21.0 ± 10.9°; *p* = 0.003). The *DHI* increased significantly from pre-operatively to post-operatively in each lumbar segment. *PI*-*LL* mismatch decreased significantly after THA (pre: 3.5°; post: 1.4°; *p* < 0.001).

**Conclusion:**

The impact of THA on the spinopelvic complex was demonstrated by significantly improved lumbar flexibility and a gain in post-operative disc height. These results illustrate the close interaction between the pelvis and the vertebral column. The investigation provides new insights into the biomechanical patterns influencing the hip–spine syndrome.

**Supplementary Information:**

The online version contains supplementary material available at 10.1007/s00264-022-05497-9.

## Introduction

The spinopelvic complex represents the interaction between the hip joint, pelvis and vertebral column and has received increased attention in recent years [1, 2]. It was demonstrated that adult spinal deformity correction influences the spinopelvic complex and might alter the acetabular orientation [3, 4]. Furthermore, the impact of spinal fusion surgery on total hip arthroplasty (THA) as a potential risk factor for dislocation was highlighted [4–10]. Not only spine surgery–related factors, but also degenerative conditions of the vertebral column were identified as risk factor for THA dislocation [11, 12]. Due to the close relation of the hip and spine, there is an emerging interest in concepts of treating patients with concurrent hip and spine pathology [13]. The influence of the hip and especially THA on the lumbar spine and its sagittal alignment remains widely unknown. To date, there have only been few studies investigating the influence of THA and its effect on post-operative sagittal spinal alignment [14, 15]. The interactions between THA, the spinopelvic complex and the lumbar spine are lacking investigations in a holistic approach. Low back pain (LBP) is a large burden for the patients and accounts for the majority of back pain [16]. Although a relevant number of patients suffer from LBP, the relations within the spinopelvic complex remain widely unknown [17]. Not only is sagittal spinal alignment a contributing factor of the evolvement of LBP, also the loss of disc height is considered influential [17–19]. The improvement of health-related quality of life due to an enhanced disc intervertebral height in patients with LBP has already been demonstrated [20]. Osteoarthritis of the hip and its related pain is associated with abnormal posture; this might contribute to the development of LBP [21, 22]. However, some studies have reported decreased LBP after THA, but the underlying mechanisms have not yet been identified [14, 23–26].

The aim of this study is to gain a better understanding of the impact of THA on lumbar lordosis in sitting and standing positions and the lumbar disc height stratified by osteochondrosis intervertebralis. Furthermore, the investigation highlights to what extent the lumbar intervertebral disc height is interrelated to the spinopelvic and spinal sagittal alignment.

## Materials and methods

Patients undergoing elective primary THA were screened for study inclusion from September 2019 to November 2020 in a tertiary reference centre. The study was approved by the institutional ethics board (EA2/142/17) and is in accordance with the Declaration of Helsinki. All patients have given their written informed consent. Exclusion criteria were defined as any form of revision THA, a history of previous spinal fusion surgery at any level, ankylosing spondylitis, osseous metastases, any neurological condition affecting the posture, simultaneously bilateral performed THA and severe hip dysplasia with subsequent femoral shortening osteotomy.

### Radiographic assessment

**Fig. 1:**
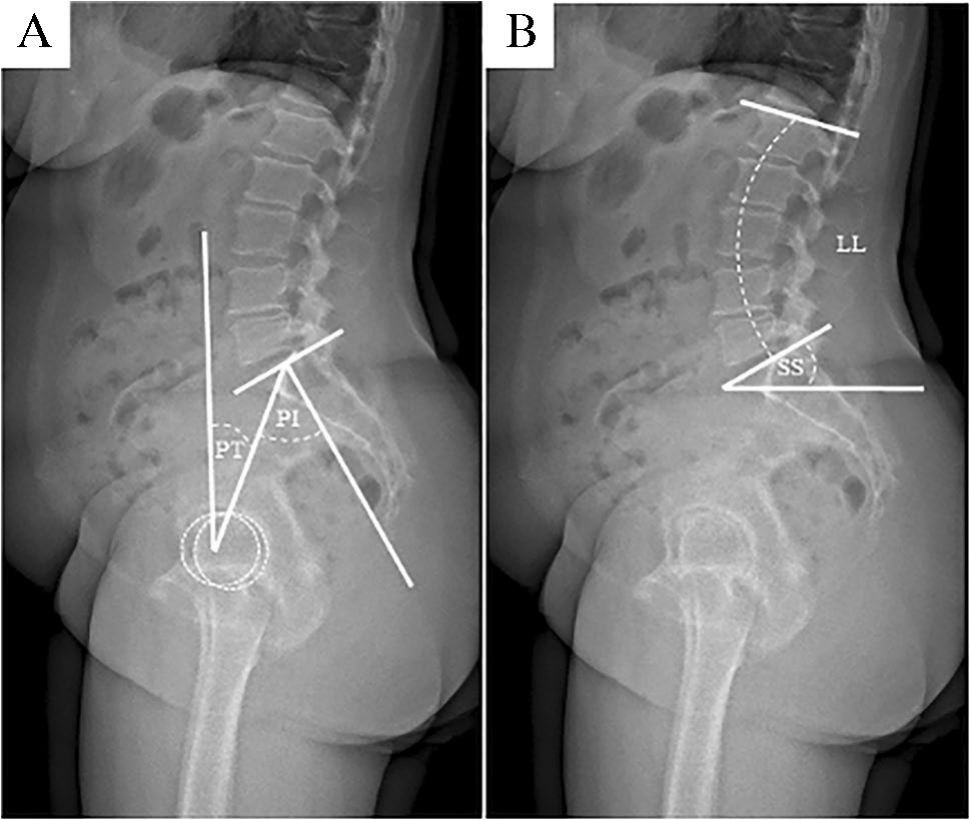
Section of a standing sagittal EOS image of the lumbar spine, pelvis and hip joints (images **A** and **B**) are depicted. Image **A** illustrates the measurement method of the pelvic tilt (*PT*) and pelvic incidence (*PI*) in the standing position. Image **B** shows the measurement of the lumbar lordosis (*LL*) and the sacral slope (*SS*) in standing position

Radiological images were obtained using biplanar low-dose stereoradiography (EOS, Paris, France) within three days pre-operatively and five to seven days post-operatively. The included patients received standing and sitting radiographs from anterior–posterior and lateral, and the entire spine was imaged up to the proximal tibia. For the standing image, the patients were asked to stand as naturally as possible and to place their hands on an arm support of the EOS device with their arms relaxed. The subjects were instructed to sit on a height-adjustable chair without a backrest, and the chair height was adjusted till the thighs were parallel to the floor. The measurements were conducted by an orthopaedic surgeon using a Merlin Diagnostic Workcenter (Phoenix-PACS, Freiburg, Germany). In addition, a randomized 25% of the dataset were re-measured by an independent orthopaedic surgeon to evaluate interrater reliability. The recorded parameters were sagittal vertical axis (*SVA*, mm), pelvic incidence (*PI*, °), lumbar lordosis (*LL*, °), pelvic tilt (*PT*, °), sacral slope (*SS*, °) and *PI*-*LL* mismatch (*PI*-*LL*, °) (Fig. 1). The differences between the standing and sitting radiographic evaluation of *LL* (∆ *LL* = *LL*_standing_ − *LL*_sitting_) were determined as lumbar flexibility.

Osteochondrosis intervertebralis was graded according to a Kellgren and Lawrence adjusted score (grades 0 to 4) [27]. The highest grade of the classification regarding all levels of the lumbar spine was determined. For reasonable comparison, the patients were then divided into groups: mild or severe osteochondrosis intervertebralis. In the mild group, patients were assigned Kellgren and Lawrence grades 0 to 2. Severe osteochondrosis intervertebralis was defined as grades 3 and 4 according to Kellgren and Lawrence.

**Fig. 2:**
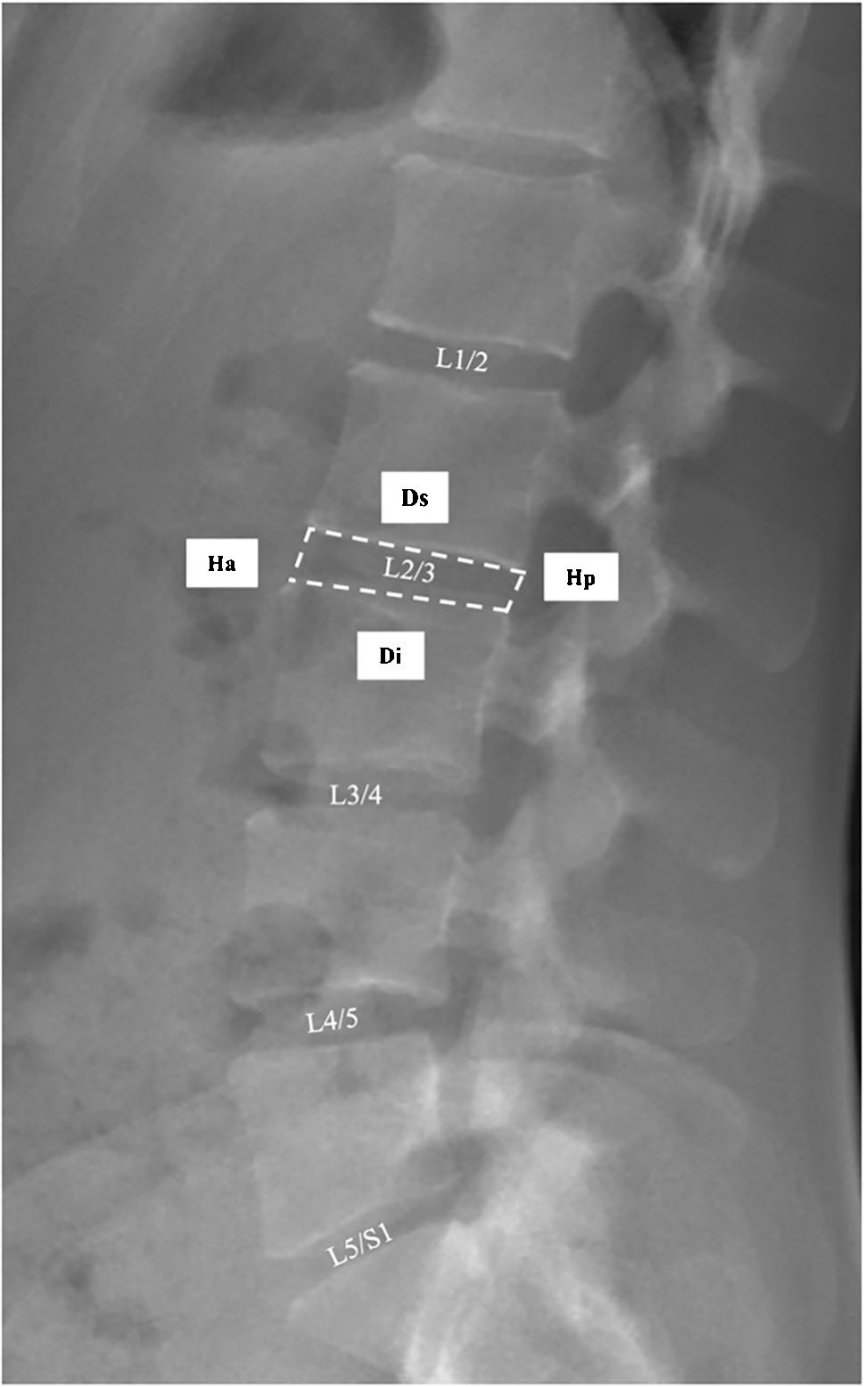
The disc height index (*DHI*) measurement method using a section of a lumbar spine standing sagittal EOS image is demonstrated The *DHI* is calculated using the disc height anterior (*Ha*), the disc height posterior (*Hp*), the disc width superior (*Ds*) and the disc width inferior (*Di*). The variables are entered into the following equation [(*Ha* + *Hp*)/(*Ds* + *Di*)] × 100. In addition, all disc compartments that were calculated are numbered (L1/2 to L5/S1) in the image shown

Measurement of the lumbar intervertebral disc height was performed according to an established method [28]. The following parameters have been collected to calculate the disc height index (*DHI*): the anterior disc height (*Ha*), the posterior disc height (*Hp*), the depth of the superior portion of the disc (*Ds*), and the depth of the inferior portion of the disc (*Di*). *DHI* was calculated based on the parameters obtained (Fig. 2).

$$\mathrm{DHI}=\left(\frac{\mathrm{Ha}+\mathrm{Hp}}{\mathrm{Ds}+\mathrm{Di}}\right)\times100$$

*DHI* was calculated for each disc compartment from L1/2 to L5/S1. A detailed description of the measured parameters is given in Supplemental Table 1.

**Table 1:** Comparison of preoperative and postoperative mean values of standing and sitting lumbar lordosis (*LL*) and the difference between standing and sitting represented by Δ*LL*. Student’s *t*-test for related samples was applied. The effect size was calculated using Cohen’s *d*. Significant *p*-values are marked in bold. Level of significance set at *p* < 0.05. *SD* standard deviation

	Preoperative	Postoperative	*p*-value	Cohen’s *d*
	Mean (± *SD*)	Mean (± *SD*)		
*LL*_sitting_ [°]	29.4 (± 15.4)	26.7 (± 15.4)	**0.01**	0.186
*LL*_standing_ [°]	51.3 (± 14.3)	52.4 (± 13.8)	** < 0.001**	0.259
Δ*LL* [°]	22.0 (± 12.3)	25.9 (± 12.3)	** < 0.001**	0.367

### Statistical analyses

Statistical analyses were performed using SPSS version 27 (IBM Corporation, NY, USA). *t*-test for connected samples were applied to compare the pre-operative with the post operative data. *t*-test for unpaired samples was applied to compare the mild and severe osteochondrosis intervertebralis groups. Pearson’s correlation was used to determine the relationship between the lumbar disc height index on each level to the spinopelvic parameter and the sagittal spinal alignment. The Spearman rank correlation coefficient was used to verify the interrater reliability of the radiological measurement. The level of significance was set as *p* < 0.05.

## Results

A total of 197 primary THA patients (106 females), with a mean age of 66.3 years (range: 17–88 years) and a mean BMI of 26.8 kg/m^2^ (range: 16.7–51.7 kg/m^2^) were eligible for analysis. The surgical indication for THA of the included patients was primary osteoarthritis of the hip (*n* = 144) and secondary osteoarthritis of the hip with *n* = 21 cases of hip dysplasia, *n* = 14 of avascular necrosis of the head, *n* = 9 of femoroacetabular impingement of the CAM type and *n* = 9 others. The results of the interrater reliability are illustrated in Supplemental Table 2.

**Table 2:** Comparison of pre-operative and post-operative mean values of standing and sitting lumbar lordosis (*LL*) and the difference between standing and sitting represented by Δ*LL* stratified by osteochondrosis intervertebralis in mild and severe according to a Kellgren and Lawrence adjusted classification. Student’s *t*-test for connected samples was performed to compare pre-operative to post-operative alterations in each group (mild or severe). Student’s *t*-test for unconnected samples was performed to compare each preoperative and postoperative data between the mild and the severe osteochondrosis groups (right table column (*p*-values M/S)). The first *p*-value represents the preoperative comparison and the second *p*-value the post-operative comparison. Significant *p*-values are marked in bold. Level of significance set at *p* < 0.05. *SD* standard deviation

	Mild osteochondrosis (*n* = 116)			Severe osteochondrosis (*n* = 81)			*p-values M/S*
	Pre	Post	*p-value*	Pre	Post	*p-value*	
	Mean (± *SD*)	Mean (± *SD*)		Mean (± *SD*)	Mean (± *SD*)		
*LL*_sitting_ [°]	29.9 (± 14.1)	26.3 (± 15.0)	**0.001**	28.7 (± 17.0)	27.2 (± 16.1)	0.166	0.620/0.714
*LL*_standing_ [°]	54.9 (± 10.5)	55.4 (± 11.1)	0.470	46.3 (± 17.2)	48.1 (± 16.0)	** < 0.001**	** < 0.001/ < 0.001**
Δ*LL* [°]	25.4 (± 11.8)	29.4 (± 12.0)	** < 0.001**	17.5 (± 11.4)	21.0 (± 10.9)	**0.003**	** < 0.001/ < 0.001**

### Changes of the lumbar lordosis after THA

*LL*_sitting_ decreased significantly (pre: 29.4 ± 15.4°; post: 26.7 ± 15.4°; *p* = 0.01), and *LL*_standing_ increased significantly after hip replacement (pre: 51.3 ± 14.3°; post: 52.4 ± 13.8°; *p* < 0.001) (Table 1). Due to the decrease in *LL*_sitting_ and the increase in *LL*_standing_, lumbar flexibility (∆*LL*) enhanced significantly to 25.9 ± 12.3°; *p* < 0.001. *PI*-*LL* mismatch decreased significantly after THA (pre: 3.5°; post: 1.4°; *p* < 0.001). The global sagittal alignment (*SVA*) did not change significantly pre- to post-operatively (pre: 53.8 mm; post: 54.6 mm; *p* = 0.68).

### Alterations of the lumbar lordosis dependent on osteochondrosis intervertebralis after THA

The mild osteochondrosis group had a mean age of 62.5 ± 13.8 years while the severe group had a mean age of 71.7 ± 9.1 years. The age difference of the two groups is statistically significant (*p* < 0.001). Lumbar flexibility improved significantly in the mild and severe osteochondrosis groups after THA. Nevertheless, there were significant differences in lumbar flexibility between the mild and severe groups, both pre-operatively and post-operatively (Table 2). *LL*_standing_ did not alter post-operatively in the mild group, while it significantly increased within the severe group. Significant differences between the mild and the severe group were demonstrated, pre-operatively and post-operatively. *LL*_sitting_ in the mild group significantly decreased after hip replacement, whereas no difference was detected in the severe osteochondrosis group. There were no significant differences in *LL*_sitting_ between the osteochondrosis groups both pre- and post-operatively.

### Disc height index

**Fig. 3:**
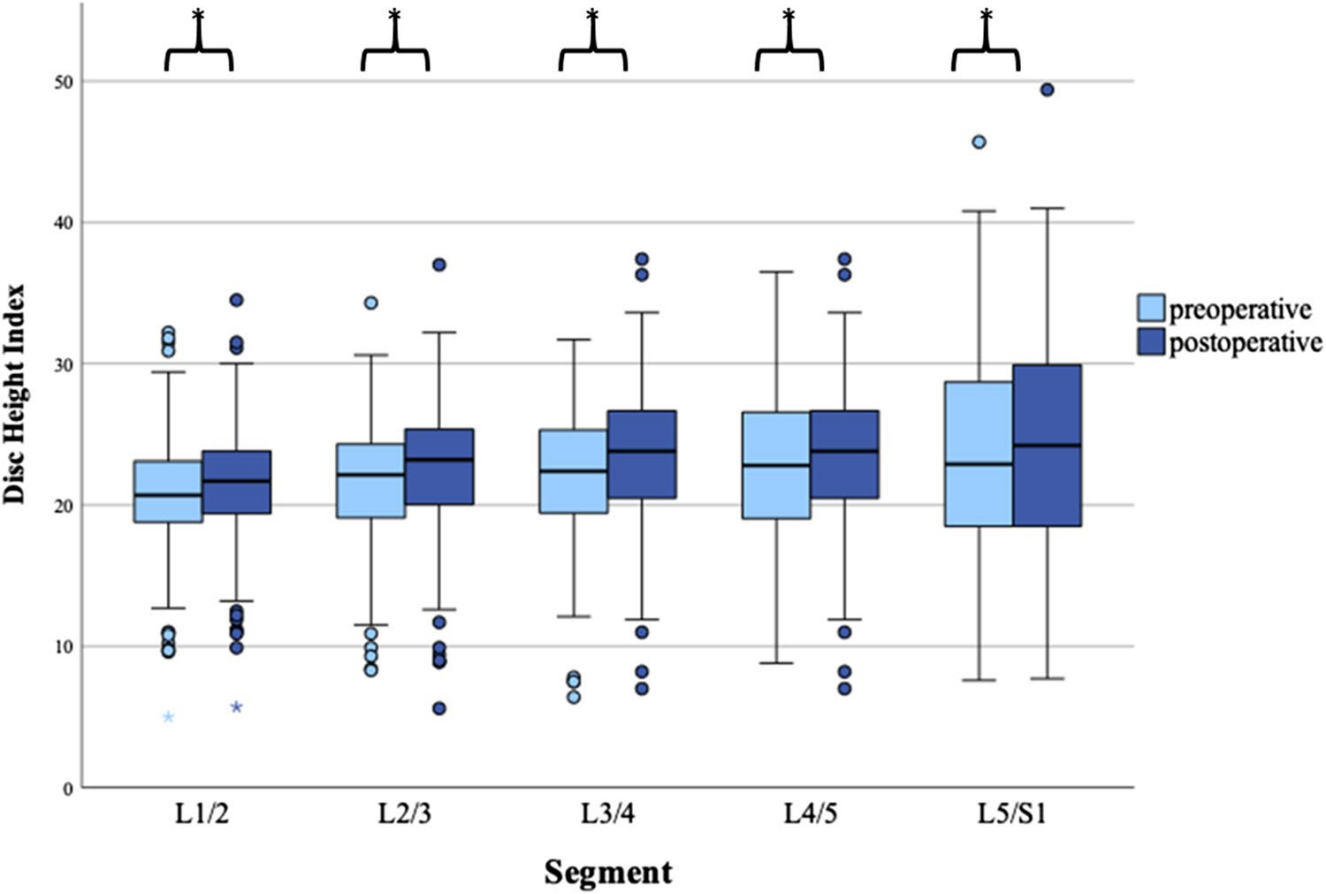
Grouped boxplots of disc height index (*DHI*) comparing pre-operative and post-operative *DHI* on each lumbar segment. Significant enhancements were detected for each individual segment for *DHI* after THA. Asterisk demonstrates significant alterations

*DHI* increased significantly in each evaluated lumbar segment after THA. *DHI* increased continuously with descending lumbar segment from 20.7 in the L1/2 segment to 23.5 in the L5/S1 segment pre-operatively, as well as post-operatively (21.5 to 24.4) (Table 3, Fig. 3).

**Table 3:** Comparison of the mean values of the individual disc height indices (*DHI*) for each lumbar spinal segment is presented. A paired sample *t*-test was used. The effect size was calculated using Cohen’s *d*. Significant *p*-values are marked in bold. The level of significance was set at *p* < 0.05

	Pre-operative	Post-operative	*p*-value	Cohen’s *d*
	Mean (± *SD*)	Mean (± *SD*)		
*DHI* L1/2	20.7 (± 4.2)	21.5 (± 4.2)	** < 0.001**	0.302
*DHI* L2/3	21.5 (± 4.5)	22.5 (± 4.7)	** < 0.001**	0.422
*DHI* L3/4	22.2 (± 4.7)	23.5 (± 4.9)	** < 0.001**	0.477
*DHI* L4/5	22.8 (± 5.7)	24.4 (± 6.3)	** < 0.001**	0.406
*DHI* L5/S1	23.5 (± 7.2)	24.4 (± 7.6)	**0.021**	0.168

### Disc height index stratified by osteochondrosis intervertebralis (mild/severe)

Lumbar *DHI* across all segments was significantly greater in the mild osteochondrosis group compared to that in the severe group, both pre- and post-operatively. There were significant increases in *DHI* from pre- to post-operatively across segments L1/2 to L4/5, with only L5/S1 revealing non-significant increases in both groups after total hip replacement (Table 4).

**Table 4:** Comparison of pre-operative and post-operative mean values of the disc height indices (*DHI*) of the individual lumbar segments stratified by osteochondrosis intervertebralis in mild and severe according to a Kellgren and Lawrence adjusted classification. Student’s *t*-test for connected samples was performed to compare pre-operative to post-operative alterations in each group (mild or severe). Student’s *t*-test for unconnected samples was performed to compare each pre-operative and post-operative data between the mild and the severe osteochondrosis groups (right table column (*p*-values M/S)). The first *p*-value represents the pre-operative comparison and the second *p*-value the post-operative comparison. Significant *p*-values are marked in bold. Level of significance set at *p* < 0.05. *SD* standard deviation

	Mild osteochondrosis (*n* = 116)			Severe osteochondrosis (*n* = 81)			*p-values M/S*
	Pre	Post	*p-value*	Pre	Post	*p-value*	
	Mean (± *SD*)	Mean (± *SD*)		Mean (± *SD*)	Mean (± *SD*)		
*DHI* L1/2	21.6 (± 3.3)	22.4 (± 3.5)	** < 0.001**	19.5 (± 5.0)	20.1 (± 4.8)	**0.028**	**0.001/ < 0.001**
*DHI* L2/3	22.5 (± 3.6)	23.5 (± 3.6)	** < 0.001**	19.8 (± 5.4)	20.9 (± 5.7)	** < 0.001**	** < 0.001/ < 0.001**
*DHI* L3/4	23.1 (± 3.9)	24.7 (± 4.0)	** < 0.001**	20.7 (± 5.3)	21.7 (± 5.6)	**0.006**	**0.001/ < 0.001**
*DHI* L4/5	23.4 (± 4.6)	25.2 (± 5.4)	** < 0.001**	22.0 (± 6.9)	22.8 (± 7.4)	**0.01**	0.113/**0.016**
*DHI* L5/S1	24.6 (± 7.1)	25.5 (± 7.4)	0.112	21.9 (± 7.0)	22.7 (± 7.6)	0.055	**0.01/0.013**

Correlation of the lumbar *DHI* with the spinopelvic complex and sagittal spinal alignment.

**Fig. 4:**
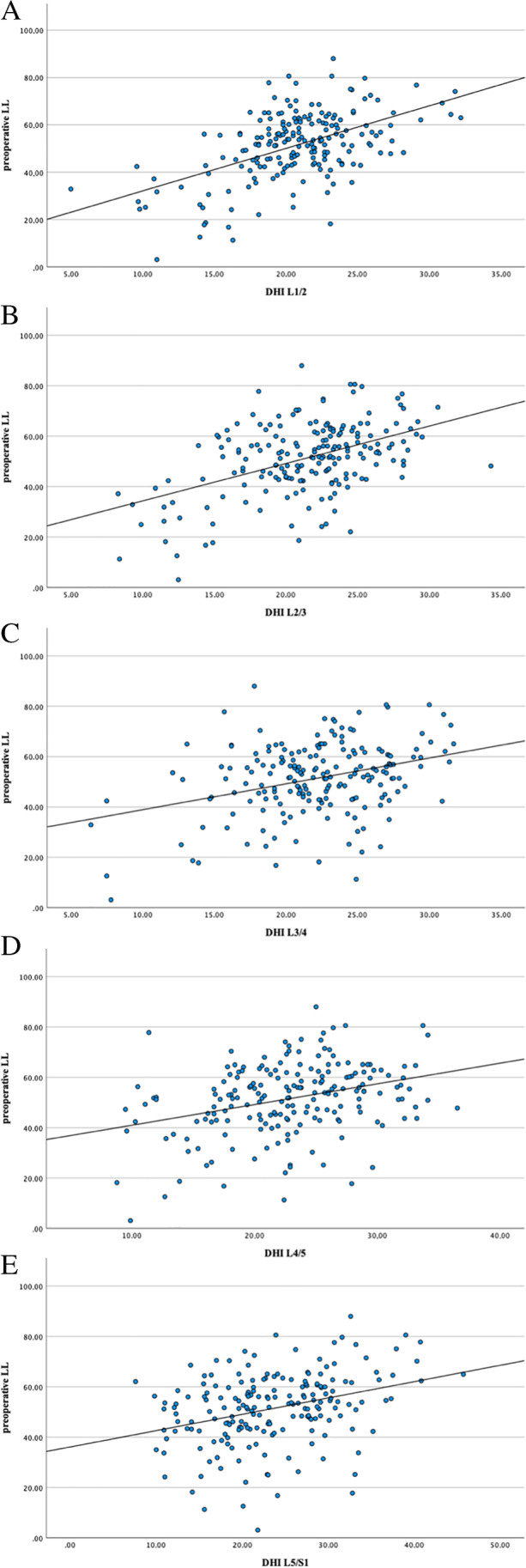
Histograms demonstrating the significant positive correlation between the disc height index (*DHI*) of the individual segments (L1/2 to L5/S1, from the top down **A**–**E**) and the preoperative lumbar lordosis in standing position (*LL*_standing_)

*LL*_standing_ revealed significant positive correlations with *DHI* in all lumbar segments (L1/2 to L5/S1) pre-operatively (Fig. 4). Lumbar flexibility (Δ*LL*) demonstrated significant positive correlations with *DHI* in all investigated segments. *SS*_standing_ was significantly positively correlated in all *DHI* segments. The sagittal spinal alignment represented by *PI*-*LL* mismatch and the *SVA* correlated significantly negative with *DHI*, except for *DHI* L4/5 (*SVA*) (Supplemental Table 3).

The post-operative correlations between *DHI* and sagittal and spinopelvic alignment are comparable to those pre-operatively. *LL*_standing_ had the highest positive correlations with the *DHI* postoperatively in all segments. Δ*LL* demonstrated significantly positive correlations with all *DHI* levels. *SS*_standing_ revealed a moderate positive correlation with all *DHI* levels post-operatively. Overall, *PI*_standing_ demonstrated no correlation with the *DHI* on different levels, both pre-operatively and post-operatively, except for L1/2 (Supplemental Table 4).

## Discussion

The aim of the prospective observational study was to evaluate the influence of THA on the lumbar disc height index and the lumbar vertebral column in different functional positions. To the best of the authors’ knowledge, this is the first study investigating the influence of THA on the lumbar intervertebral disc height and demonstrating a significant enhancement of the lumbar disc height index postoperatively, even in patients classified with severe osteochondrosis.

The influence of adult spine deformity reconstruction on the spinopelvic complex has already been demonstrated [29–31]. Thereby, it was demonstrated that re-storing of the sagittal spinal alignment might led to a decrease in posterior pelvic tilt and acetabular anteversion [4, 32, 33]. However, only a few investigations assessed the influence of hip replacements on the spinopelvic complex [14, 22, 34]. Weng et al. examined the changes in spinopelvic alignment after THA in a patient cohort with confirmed LBP demonstrating a significant alteration in T1 spinal–pelvic inclination, pelvic–femoral angle and a significantly reduced prevalence of LBP post-operatively [14]. Interestingly, no significant changes of *LL*_standing_ were detected in their investigation, contrasting our results. The distinct smaller patient populations in their study compared to that in our investigation might have been a reason, whereas Jain et al. revealed an impact of THA with a significant decrease of sagittal spinal malalignment and significantly altered spinopelvic parameters [22]. Besides Weng et al., other studies obtained similar findings resulting in improved LBP after THA [23, 25, 35]. However, despite obvious clinical evidence, the exact mechanism of THA-related LBP reduction has not been clarified yet. A conceivable factor might be the release of capsular and muscle contractures of the hip joint, occurring with severe osteoarthrosis. Nevertheless, recently Okuzu et al. identified preoperative factors associated with the reduction of LBP after THA [36]. A small Cobb angle was associated with a post-operative decrease of LBP to reduce, whereas sagittal spinal imbalance and a higher Cobb angle were identified as risk factors for persistent LBP [36]. It is known that a restriction in an individual segment of the spinopelvic complex, such as restricted pelvic mobility, is compensated within other segments in the spinopelvic complex [37]. Our results demonstrated enhanced lumbar lordosis in the sitting position and decreased *LL* in the standing position pre-operatively, which might highlight a compensation mechanism for capsular and muscular contractures associated with severe osteoarthritis of the hip. The greater lordosis in the sitting position might lead to a mechanical misloading of the lumbar spine and thus can promote the development of LBP [38]. Following the assumption, the release of capsular and muscle contractures by THA enables an improved spinopelvic interaction. Consistent with this hypothesis, improved pelvic mobility after THA was demonstrated previously [39].

Consequently, our results revealed a significant reduction in sitting *LL* and a significant increase in *LL*_standing_ and lumbar flexibility post-operatively. These improvements are even observed in patients with severe osteochondrosis intervertebralis, highlighting the possible influence of THA on the lumbar spine. Buckland et al. demonstrated in patients with severe hip osteoarthritis a reduced range of motion of the hip joint, which was compensated for by other segments of the spinopelvic complex [2]. They also highlighted the relationship between severe osteoarthritis of the hip and increased *LL*_sitting_ in patients with restricted pelvic mobility [2]. In line with their findings is the significantly altered Δ*LL* in our investigation.

Another possible hypothesis for the improvement in lumbar flexibility after THA might be the pain due to progressed osteoarthritis of the hip. As the source of pain was treated by the THA, post-operative posture adaptions might took place. There might be a possible relationship between hip-related pain and restrictions of the spinopelvic motion. In addition to the post-operative improvement in sagittal spinal (*PI*-*LL*) and spinopelvic (Δ*LL*, *LL*_standing_, *LL*_sitting_) alignments, the lumbar disc height was enhanced significantly above all lumbar segments after THA, even in the severe osteochondrosis subgroup. Our results suggest that THA might have a positive impact on LBP through increased lumbar disc height, more physiological lumbar lordosis in standing and sitting and an enhanced lumbar flexibility. These findings may provide crucial missing explanatory patterns for the hip–spine syndrome, which is not yet fully understood. Our assumption that an enhancement of lumbar intervertebral *DHI* is related to a reduction in LBP is in line with the results of Lidar et al. [20]. They demonstrated an increase of the intervertebral disc height on level L4–L5 (pre: 6 ± 1 mm; post: 8 ± 1 mm) one year after bariatric surgery and a subsequent significant reduction of LBP (VAS pre: 5.70 ± 3.12; post: 1.33 ± 2.13) in their patient collective [20].

The reduction of *PI*-*LL* mismatch is due to the significant alterations of *LL* post-operatively and underlines the close interaction between the pelvis and lumbar spine. Some studies reported a reduction in LBP after hip replacement; so far, a conclusive biomechanical and clinical explanation is lacking [14, 23, 26]. LBP is known to be related to sagittal spinal alignment, suggesting a relation to the detected decrease in *PI*-*LL* mismatch in our investigation to the reported LBP relief after THA [17]. The postoperative intervertebral disc height enhancements and the increase of lumbar flexibility might support the understanding of the biomechanical interactions between THA and the relief of LBP, often referred to as hip–spine syndrome [40].

The study has several limitations which need to be considered. The radiographic EOS imaging demonstrates results from a short-term follow-up. It needs to be taken into account that the posture may have been influenced by surgery-related pain. However, each THA patient received standardized and individual adapted stepwise pain management. Following this, it can be assumed that the short follow-up had no relevant influence on the posture. Confirming our short-term follow-up results, a long-term follow-up is planned. We do not assume a relevant influence of the severity of the contralateral hip osteoarthritis on the lumbar spine, since around 70% (*N* = 107) of the analysed patients had only a mild hip osteoarthritis (Kellgren and Lawrence grades 1 and 2) of the contralateral side (Supplemental Table 5). Nevertheless, the impact of severe hip osteoarthritis on the lumbar spine cannot be completely ruled out. In addition, the influence of a pre-existing contralateral THA on the lumbar vertebral column cannot be completely excluded. Even though the discussion of lumbar disc height changes after THA is affiliated to LBP, it should be noted that low back pain was not documented in our patient collective, and our investigation is lacking clinically relevant data regarding LBP. Another point worth mentioning is that despite the significant improvement in lumbar flexibility, to date there is no minimum clinically important difference defined. Therefore, we cannot conclusively determine whether the enhancement in lumbar flexibility is clinically meaningful. It should be noted that spinal disc height can vary with the time of day, which might influence the results.

## Conclusion

To the best of the authors’ knowledge, this is the first study confirming a significant enhancement of lumbar disc height and lumbar flexibility and alteration of the lumbar alignment in sitting and standing position after THA. We demonstrated that the influence of THA through the spinopelvic interactions on the lumbar spine extends far beyond simple osteoarthritis of the hip. This study was able to highlight the complex interaction of the hip joint, pelvis and lumbar spine in the context of THA to gain a better understanding of the patterns leading to the hip–spine syndrome.

## Author contribution

Z.H. — Formal analysis, data collection.

S.H. — Revised manuscript, supervised investigation.

M.P. — Conceptualization, revised manuscript, supervised investigation,

L.B. — Conceptualization, measurements, revised manuscript, provided graphic, supervised investigation.

H.H. — Conceptualization, measurements, data collection, supervised investigation, revised manuscript.

All authors have read and agreed to the published version of the manuscript.

## Supplementary Information

Below is the link to the electronic supplementary material.

Supplementary file1 (DOCX 905 KB)

## Data Availability

The datasets generated and analysed during the current study are available from the corresponding author on reasonable request.
