# Supplementary material for: Hip replacement improves lumbar flexibility and intervertebral disc height — a prospective observational investigation with standing and sitting assessment of patients undergoing total hip arthroplasty
Source: Int Orthop. 2022 Jul 11;46(10):2195–203. doi: 10.1007/s00264-022-05497-9 (PMC9492615; doi:10.1007/s00264-022-05497-9)
Supplement: Supplementary file 1 — Supplementary file1 (DOCX 905 KB) [file 264_2022_5497_MOESM1_ESM.docx]

## Supplements

**Radiological Parameter**

| **Radiological Parameter** | **Description** |
| --- | --- |
| Sagittal vertical axis (SVA) | Distance measured in millimeters horizontally from the C7 plumb line to the posterior edge of the superior sacral endplate. |
| Lumbar lordosis (LL) | Angle between superior endplate of L1 and superior endplate of S1 |
| Pelvic incidence (PI) | Angle between the line connecting the midpoint of the superior plate of S1 and the midpoint of the hip axis with the line perpendicular to the superior plate of S1. |
| PI – LL Mismatch (PI-LL) | Calculated as the difference of pelvic incidence (PI) minus lumbar lordosis (LL) |
| Sacral slope (SS) | Angle between the superior endplate of S1 and a horizontal line. |
| Pelvic tilt (PT) | Angle between the line joining the midpoint of the hip axis to the midpoint of S1superior endplate and the vertical reference line |
| Disc height Index (DHI) | Anterior and posterior disc height (Ha & Hp) and superior and inferior disc depth (Ds & Di) are measured in the lateral radiograph and then inserted into the following formula: ((Ha + Hp)/(Ds + Di)) × 100 |

**Supplement Table 1**. Measured radiological spinopelvic parameters and global spinal parameters as well as the disc height index (DHI) with detailed information on how these different parameters are measured.

**Interrater reliability**

|  | *Preoperative* | *Postoperative* | *Mean (Pre-postoperative)* | |
| --- | --- | --- | --- | --- |
| Sagittal Vertical Axis (SVA) | 0.614 | 0.571 | | 0.593 |
| Lumbar lordosis (LL_stand_) | 0.746 | 0.779 | | 0.763 |
| Pelvic incidence (PI_stand_) | 0.534 | 0.689 | | 0.612 |
| Pelvic tilt (PT_stand_) | 0.900 | 0.816 | | 0.858 |
| Sacral Slope (SS_stand_) | 0.533 | 0.711 | | 0.622 |
| DHI L1/2 | 0.437 | 0.377 | | 0.407 |
| DHI L2/3 | 0.503 | 0.384 | | 0.444 |
| DHI L3/4 | 0.597 | 0.529 | | 0.563 |
| DHI L4/5 | 0.408 | 0.359 | | 0.384 |
| DHI L5/S1 | 0.437 | 0.488 | | 0.463 |

**Supplement Table 2.** Pre-and postoperative values and the mean of interrater reliability of the global spinal alignment and spinopelvic parameters. Global spinal alignment and spinopelvic parameters are measured in standing position. Spearman´s rank correlation coefficient was used.

| **Preoperative Disc Height Index compared to sagittal spinal and spinopelvic Alignment** | | | | | | |
| --- | --- | --- | --- | --- | --- | --- |
|  |  | DHI  L1/2 | DHI  L2/3 | DHI  L3/4 | DHI  L4/5 | DHI  L5/S1 |
| SVA [mm] |  | **-0.206*** | **-0.308**** | **-0.266**** | -0.094 | **-0.207**** |
| PI-LL [°] |  | **-0.327**** | **-0.455**** | **-0.362**** | **-0.321**** | **-0.237**** |
| PI _stand_ [°] |  | **0.258**** | 0.067 | 0.006 | 0.039 | 0.126 |
| LL_stand_ [°] |  | **0.528**** | **0.474**** | **0.335**** | **0.328**** | **0.330**** |
| ΔLL [°] |  | **0.278**** | **0.372**** | **0.329**** | **0.220**** | **0.240**** |
| PT_stand_ [°] |  | -0.091 | **-0.254**** | **-0.258**** | **-0.231**** | **-0.158*** |
| ΔPT [°] |  | -0.092 | **-0.188**** | **-0.193**** | -0.126 | -0.059 |
| SS_stand_ [°] |  | **0.391**** | **0.287**** | **0.223**** | **0.237**** | **0.288**** |
| ΔSS [°] |  | 0.131 | **0.153*** | **0.179*** | 0.096 | 0.110 |

**Supplement Table 3.** The correlation between preoperative disc height index (DHI) for each individual lumbar segment and sagittal vertical axis (SVA), PI-LL Mismatch (PI-LL), pelvic incidence (PI), lumbar lordosis (LL), pelvic tilt (PT) and sacral slope (SS) as well as ΔLL, ΔPT and ΔSS was evaluated. Significant values are marked in bold. r-values marked with * are significant at the 0.05 level. r-values marked with ** are significant at the 0.01 level. Pearson correlation coefficient was used.

| **Postoperative Disc Height Index compared to sagittal spinal and spinopelvic Alignment** | | | | | | |
| --- | --- | --- | --- | --- | --- | --- |
|  |  | DHI  L1/2 | DHI  L2/3 | DHI  L3/4 | DHI  L4/5 | DHI  L5/S1 |
| SVA [mm] |  | **-0.258**** | **-0.282**** | **-0.268**** | **-0.190**** | **-0.240**** |
| PI-LL [°] |  | **-0.261**** | **-0.404**** | **-0.381**** | **-0.357**** | **-0.291**** |
| PI _stand_ [°] |  | **0.236**** | 0.024 | -0.012 | 0.014 | 0.048 |
| LL_stand_ [°] |  | **0.457**** | **0.394**** | **0.337**** | **0.340**** | **0.315**** |
| ΔLL [°] |  | **0.309**** | **0.445**** | **0.410**** | **0.204**** | **0.273**** |
| PT_stand_ [°] |  | -0.054 | **-0.274**** | **-0.258**** | **-0.208**** | **-0.190**** |
| ΔPT [°] |  | -0.139 | **-0.298**** | **-0.274**** | **-0.146*** | -0.100 |
| SS_stand_ [°] |  | **0.343**** | **0.271**** | **0.212**** | **0.214**** | **0.240**** |
| ΔSS [°] |  | **0.156*** | **0.271**** | **0.232**** | 0.088 | 0.139 |

**Supplement Table 4.** The correlation between postoperative disc height index (DHI) and sagittal vertical axis (SVA), PI-LL mismatch (PI-LL), pelvic incidence (PI), lumbar lordosis (LL), pelvic tilt (PT) and sacral slope (SS) as well as ΔLL, ΔPT and ΔSS was evaluated. Significant values are marked in bold. r-values marked with * are significant at the 0.05 level. r-values marked with ** are significant at the 0.01 level. Pearson correlation coefficient was used.

| **Osteoarthritis of the contralateral hip joint** | | | |
| --- | --- | --- | --- |
| *Kellgren and Lawrence grades* | *Quantity (N)* | *Proportion (%)* | *Age (years)*  *Mean*±SD |
| 0 | 0 | 0 | - |
| 1 | 2 | 1.30 | 71.5±7.8 |
| 2 | 105 | 68.62 | 65.4±12.8 |
| 3 | 29 | 18.95 | 65.8±11.5 |
| 4 | 17 | 11.11 | 62.6±17.6 |
| Pre-existing THA | 44 |  | 69.8±11.8 |

**Supplement Table 5.** Severity of the osteoarthritis of the contralateral hip joint in the control group by using the Kellgren and Lawrence system for the classification of the severity of osteoarthritis. Number of patients with existing total hip arthroplasty on the contralateral side. The portion is calculated from patients without pre-existing THA. Mean and standard deviation (SD) is presented for the patient’s age.

*
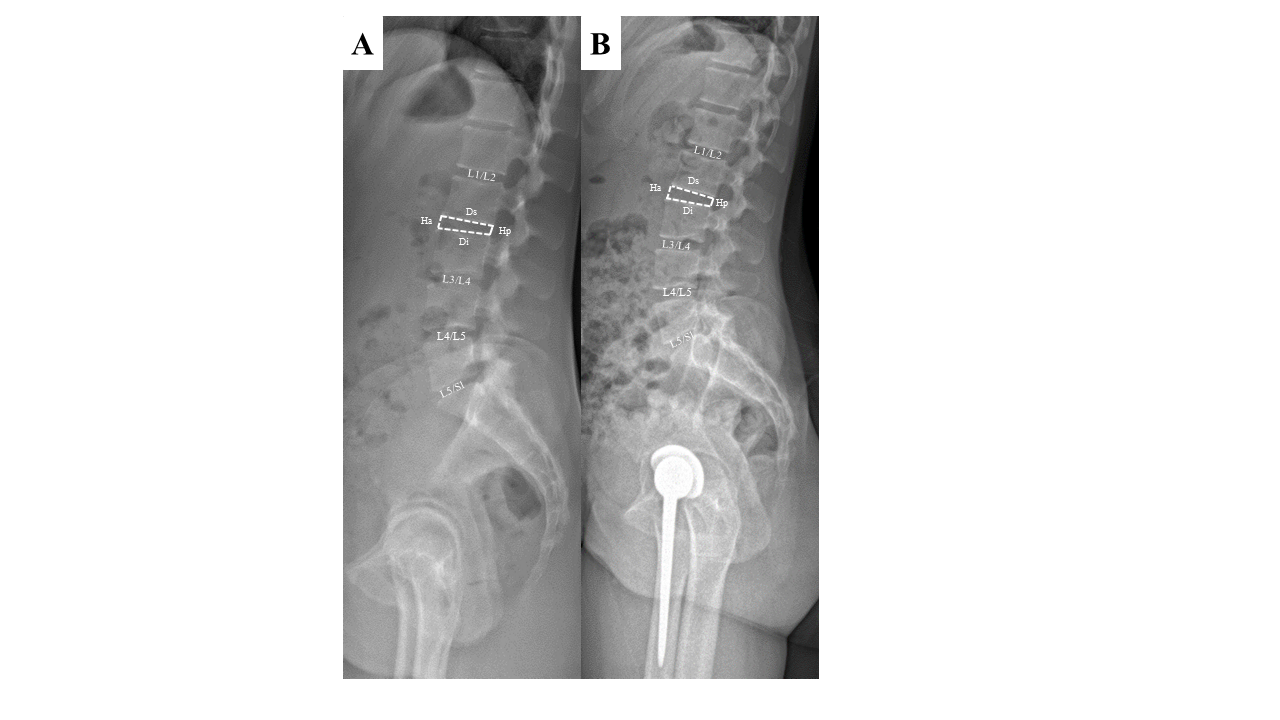
*

**Supplement Figure 1.** Measurement of disc height index pre and postoperatively in the same patient. Figure A demonstrates the measurement of the Disc Height Index (DHI) of the L2/L3 segment preoperatively. Figure B shows the measurement of the DHI of L2/L3 in the same patient postoperatively. The variables are entered into the following equation: [(Ha+Hp)/(Ds+Di)] x 100 to obtain the DHI.

*
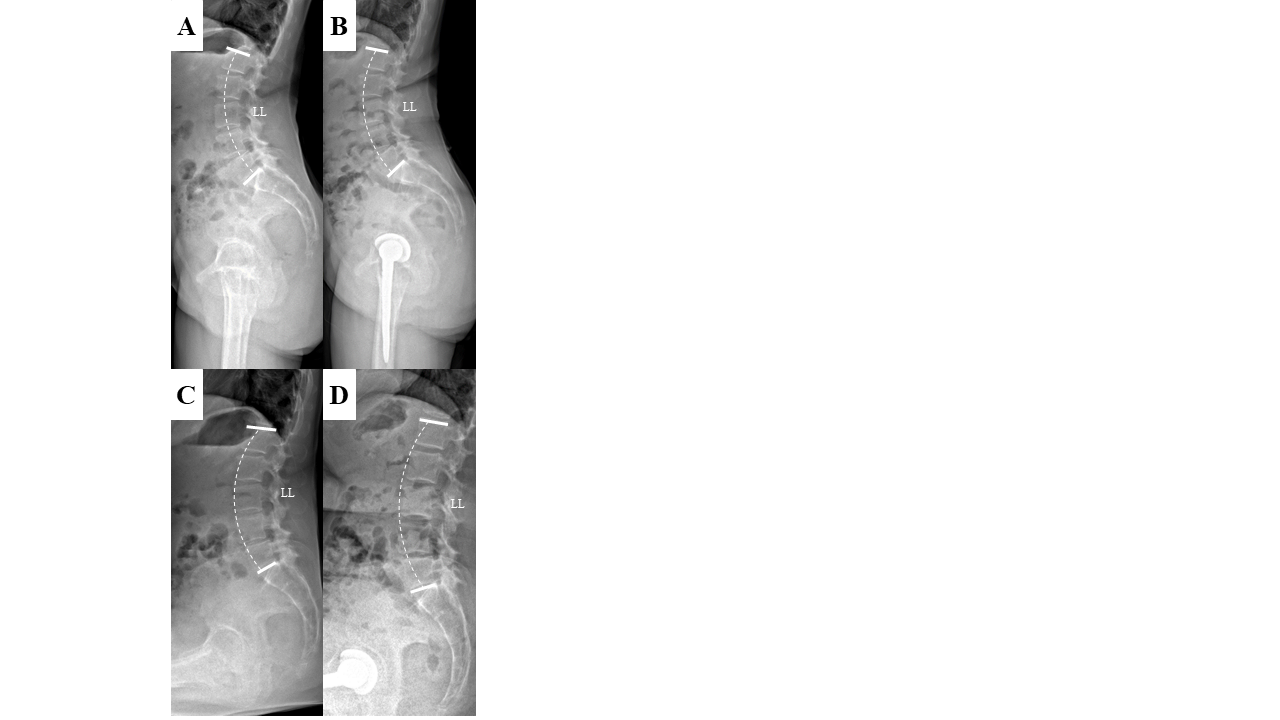
*

**Supplement Figure 2. Change in lumbar lordosis from pre to postoperative standing and sitting.** Figure A shows the lumbar lordosis (LL) in the standing position preoperatively. Picture B demonstrates the LL in standing postoperatively after total hip arthroplasty. Picture C displays the preoperative LL in sitting position. Figure D shows the LL in the sitting position postoperatively.
